# Differential effects of *Radix Paeoniae Rubra *(*Chishao*) on cytokine and chemokine expression inducible by mycobacteria

**DOI:** 10.1186/1749-8546-6-14

**Published:** 2011-03-30

**Authors:** Liangjie Wang, Cindy Lai Hung Yang, Terry Cho Tsun Or, Gang Chen, Jian Zhou, James Chun Bong Li, Allan Sik Yin Lau

**Affiliations:** 1 Molecular Chinese Medicine Laboratory, Li Ka Shing Faculty of Medicine, The University of Hong Kong, Pokfulam, Hong Kong SAR, PR China; 2 Cytokine Biology Group, Department of Paediatrics and Adolescent Medicine, The University of Hong Kong, Pokfulam, Hong Kong SAR, PR China; 3 Department of Pharmacology and Pharmacy, The University of Hong Kong, Pokfulam, Hong Kong SAR, PR China; 4 Purapharm International Hong Kong, Central, Hong Kong SAR, PR China

## Abstract

**Background:**

Upon initial infection with mycobacteria, macrophages secrete multiple cytokines and chemokines, including interleukin-6 (IL-6), IL-8 and tumor necrosis factor-α (TNF-α), to mediate host immune responses against the pathogen. Mycobacteria also induce the production of IL-10 *via *PKR activation in primary human monocytes and macrophages. As an anti-inflammatory cytokine, over-expression of IL-10 may contribute to mycobacterial evasion of the host immunity. *Radix Paeoniae Rubra *(RPR, *Chishao*), a Chinese medicinal herb with potentials of anti-inflammatory, hepatoprotective and neuroprotective effects, is used to treat tuberculosis. This study investigates the immunoregulatory effects of RPR on primary human blood macrophages (PBMac) during mycobacterial infection.

**Methods:**

The interaction of Bacillus Calmette-Guerin (BCG) with PBMac was used as an experimental model. A series of procedures involving solvent extraction and fractionation were used to isolate bioactive constituents in RPR. RPR-EA-S1, a fraction with potent immunoregulatory effects was obtained with a bioactivity guided fractionation scheme. PBMac were treated with crude RPR extracts or RPR-EA-S1 before BCG stimulation. The expression levels of IL-6, IL-8, IL-10 and TNF-α were measured by qPCR and ELISA. Western blotting was used to determine the effects of RPR-EA-S1 on signaling kinases and transcriptional factors in the BCG-activated PBMac.

**Results:**

In BCG-stimulated macrophages, crude RPR extracts and fraction RPR-EA-S1 specifically inhibited IL-10 production while enhanced IL-8 expression at both mRNA and protein levels without affecting the expressions of IL-6 and TNF-α. Inhibition of BCG-induced IL-10 expression by RPR-EA-S1 occurred in a dose- and time-dependent manner. RPR-EA-S1 did not affect the phosphorylation of cellular protein kinases including MAPK, Akt and GSK3β. Instead, it suppressed the degradation of IκBα in the cytoplasm and inhibited the translocation of transcription factor NF-κB1 p50 to the nucleus.

**Conclusion:**

RPR crude extracts and its fraction RPR-EA-S1 inhibited anti-inflammatory cytokine IL-10 and enhanced pro-inflammatory chemokine IL-8 expression in BCG-activated PBMac. The inhibitory effects of RPR-EA-S1 on IL-10 expression in BCG-activated PBMac may be due to the reduced nuclear translocation of NF-κB1 p50.

## Background

Tuberculosis (TB) remains a major cause of morbidity and mortality worldwide as a result of *Mycobacterium tuberculosis *(Mtb) infection. In 2008, an estimated 9.4 million new cases of TB and 1.3 million deaths were recorded globally [1]. Although anti-TB drugs and vaccines have been used for decades, the emergence of multidrug-resistant TB (MDR-TB) and the co-infection of Mtb and related mycobacteria with human immunodeficiency virus (HIV) pose new challenges to the treatment of TB [1,2].

Upon Mtb infection, multiple immune cells including macrophages/monocytes [3], dendritic cells (DCs) [4], neutrophils [5], natural killer cells [6] and T cells [6] are activated to mediate host defense against the pathogen. Among these cell types, macrophages are the main immunocytes in initiating innate immunity against Mtb. Macrophages are professional antigen presenting cells that are important in bridging innate and adaptive immunity [7]. Macrophages exert their direct anti-microbial effects through phagocytosis [8], generation of reactive oxygen intermediates (ROIs) and reactive nitrogen intermediates (RNIs) [9], autophagy [10], activation of vitamin D pathway [11], apoptosis [12] and secretion of cytokines and chemokines [13].

Specific cytokines and chemokines produced by macrophages during Mtb infection play important roles in regulating the host innate and adaptive immune responses [13]. For example, tumor necrosis factor-α (TNF-α) enhances the activity of macrophage to kill replicating Mtb in synergy with interferon-γ (IFN-γ) [14] and contributes to the formation of granuloma and prevention of mycobacterial dissemination through decreased cell migration [15]. Another key cytokine is interleukin-6 (IL-6), which is a pro-inflammatory cytokine that regulates B and T cell activities and contributes to the initial innate responses to Mtb [16].

IL-8, another important chemokine against Mtb, is primarily secreted by monocytes and macrophages but also produced by fibroblasts, keratinocytes, and lymphocytes [17]. The induction of IL-8 can be stimulated by multiple factors including pathogens, toxins and cytokines [18]. Previous studies have demonstrated the connection between IL-8 production and Mtb infection. In clinical studies, remarkably elevated levels of IL-8 were found in tuberculous pleural exudate, bronchoalveolar lavage fluid, cerebrospinal fluid and tuberculous granulomas [19]. In contrast, decreased IL-8 secretion was observed in HIV-infected patients with miliary TB, suggesting that the absence of IL-8 induction is detrimental to restricting Mtb dissemination [20]. Such important role of IL-8 was illustrated in a previous study that upon Mtb infection IL-8 attracted neutrophils and T lymphocytes to the infection sites at an early stage, resulting in the formation of granuloma [21].

Unlike the pro-inflammatory cytokines IL-6 and IL-8, interleukin-10 (IL-10) is a key anti-inflammatory cytokine produced by macrophages, Th1 cells [22], Th2 cells [22], regulatory CD4^+ ^T cells [23], CD8^+ ^T cells [23], DCs and B cells [24]. IL-10 mainly plays a negative role in the regulation of immunity to prevent uncontrolled responses to pathogens [25,26]. It inhibits the production of pro-inflammatory cytokines, such as IFN-γ, IL-12, IL-18, IL-1 and TNF-α, by macrophages and DCs [27]. Together with reduced induction of inducible nitric oxide synthases (iNOS) and ROIs, IL-10 expression results in reduced killing of intracellular pathogens by macrophages [28]. IL-10 is also an inhibitor of antigen presentation by macrophages and DCs through down-regulation of MHCII expression [29-31]. In the event of TB progression, over-expression of IL-10 results in a higher mycobacterial burden and reactivation of pulmonary TB [32]. Macrophages from IL-10 deficient mice, when challenged with BCG, produce higher levels of TNF-α with concomitant accelerated clearance of mycobacteria [33]. These observations were further validated by clinical studies, showing higher levels of IL-10 production in patients with active pulmonary TB [34,35]. Moreover, overproduction of IL-10 by T cells was shown to be related to suppressed immunity and increased susceptibility to mycobacteria infection [36].

*Radix Paeoniae Rubra *(RPR, *Chishao*), the dried root of *Paeonia lactiflora *Pallas or *Paeonia veitchii *Lunch [37], has been widely used by Chinese medicine practitioners to treat cardiovascular, inflammation and female reproductive diseases [38]. Based on the principle of Chinese medicine, historical literatures described RPR with the functions of tonifying blood, cooling blood, cleansing heat, and invigorating blood circulation. It is often used as an "Assistant" herb to counteract or ameliorate the undesirable side effects of the "King" herbs [37]. In contemporary literatures, RPR is used for the treatment of the following diseases: seasonal febrile diseases with eruptions, bleeding, menstrual disorders, trauma, skin infection and conjunctivitis as well as pain over the chest, hypochondrium and abdomen [39]. RPR is also used in some herbal formulae to treat TB patients [40]. RPR has protective effects on lung injury through the induction of heme oxygenase-1 (HO-1) and suppression of nitric oxide (NO) in rats [41,42]. In a mouse model, the extracts obtained from RPR and *Astragalus membranaceus *protected the liver from BCG/endotoxin-induced injury. These protective effects were associated with downregulation of pro-inflammatory cytokines [43]. While some studies on the biological effects and chemical constituents of RPR have been reported [38], the immunoregulatory effects of RPR and their detailed mechanisms of action at cellular signaling levels are yet to be investigated, especially the interaction of BCG with human blood macrophages.

In the present study, an active fraction, namely RPR-EA-S1, was isolated from the crude extracts of RPR using a bioactivity-guided fractionation scheme. The chemical profile of RPR-EA-S1 was analyzed by high performance liquid chromatography (HPLC) and gas chromatography mass spectrometry (GC-MS). The regulatory effects of crude RPR extract and its fraction RPR-EA-S1 on BCG-induced cytokine and chemokine expression in human PBMac were examined. The underlying mechanisms of the inhibitory effects of RPR-EA-S1 on BCG-induced IL-10 expression were delineated.

## Methods

### Plant material

*Radix Paeoniae Rubra *was obtained from PuraPharm International Ltd. (Hong Kong, China). The raw material of the herb was authenticated by PuraPharm Corporation (Guangxi, China) according to the method in the Pharmacopoeia of the People's Republic of China 2005 [44] which states that the RPR sample should not contain less than 1.8% of paeoniflorin, calculated with reference to the dried substance. The *Radix Paeoniae Rubra *used in our study contained more than 4% of paeoniflorin.

### Preparation of the crude extract from *Radix Paeoniae Rubra*

**Figure 1:**
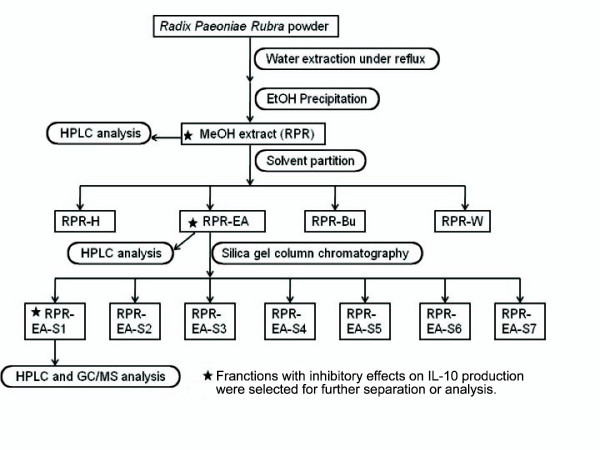
**Extraction and separation scheme of RPR-EA-S1 from RPR**. RPR powder was extracted by Milli Q water under reflux and the extract was precipitated with EtOH. The resulting supernatant was concentrated and partitioned sequentially with *n*-C_6_H_14_, EtOAc and *n*-BuOH. The extracts were tested for their inhibitory effects on IL-10 production in primary human blood macrophages stimulated by BCG. The bioactive RPR-EA was further separated by silica gel column chromatography. RPR-EA-S1 fraction was selected by bioactivity assay and analyzed by HPLC and GC-MS.

The procedures for raw herb extraction and fractionation were shown in Figure 1. Briefly, the herbs (300 g) were ground into powder and extracted with eight folds (volume) of Milli Q water (Sartorius, Germany) under reflux for one hour. The extraction was repeated for three times. The resulting extracts were combined and concentrated at 70°C under reduced pressure until the density reached 1.28 g/mL. Then the paste collected was precipitated with five folds (volume) of ethanol (EtOH). After filtration, the supernatant was evaporated to dryness in an evaporator (Rotavapor R-200, BUCHI, Switzerland), yielding crude RPR extract (81 g). The extract was then dissolved in methanol (MeOH) and partitioned with an equal volume of hexane (*n*-C_6_H_14_) for three times. The MeOH extract was dried and re-dissolved in water and then partitioned sequentially with an equal volume of ethyl acetate (EtOAc) and *n*-butanol (*n*-BuOH) for four times. Four fractions were obtained, namely RPR-H, RPR-EA (10 g), RPR-Bu and RPR-W. According to a bioassay guided scheme, the fraction RPR-EA with the most potent activity on IL-10 suppression was selected and further separated by silica gel column chromatography (Merck, Germany). After loading the sample, the column was washed sequentially with a series of solvents: 50% *n*-C_6_H_14 _in EtOAc, 100% EtOAc, EtOAc combined with 10%, 30%, 50% or 70% of MeOH and finally 100% MeOH. These serial washings yielded seven fractions: RPR-EA-S1 (0.78 g), RPR-EA-S2, RPR-EA-S3, RPR-EA-S4, RPR-EA-S5, RPR-EA-S6 and RPR-EA-S7.

### Chemicals and antibodies

All the chemical solvents such as EtOH, MeOH, *n*-BuOH, EtOAc and *n*-C_6_H_14 _were purchased from Merck (Germany). Antibodies against NF-κB1 p50, actin, IκBα and Lamin B were purchased from Santa Cruz Biotechnology (USA). Antibodies against phospho-ERK1/2, phospho-p38, phospho-Akt and phospho-GSK3β as well as antibodies against total ERK1/2, p38, Akt and GSK3β were purchased from Cell Signaling Technology (USA). Anti-rabbit IgG HRP-conjugated secondary antibodies were purchased from BD Biosciences (USA). Anti-goat IgG HRP-conjugated secondary antibodies were purchased from ZYMED Laboratory (USA).

### Bacillus Calmette-Guerin

Bacillus Calmette-Guerin (BCG) is a live freeze-dried vaccine made from an attenuated strain of *Mycobacterium bovis*, Danish strain 1331. The vaccine, which contains no virulent Mtb, was purchased from Statens Serum Institut (Denmark).

### Isolation of primary human peripheral blood macrophages

Using Ficoll-Paque (GE Healthcare, USA) density gradient centrifugation as previously described [3,29], we isolated primary human peripheral blood macrophages (PBMac) from buffy coats of healthy blood donors obtained from the Hong Kong Red Cross Blood Transfusion Service. Briefly, the fresh blood samples were centrifuged at 3000 rpm (1811*g*, Eppendorf AG, Germany) for 20 minutes and separated into plasma and blood cell layers. The plasma layer was carefully removed and heat-inactivated for 30 minutes at 56°C in water bath, followed by chilling on ice for ten minutes. It was centrifuged at 4000 rpm (3220*g*, Eppendorf AG, Germany) for ten minutes to remove the precipitates. The clear supernatant was then filtered through 0.45 μm membranes and later used as the autologous plasma added to culture macrophages. The blood cell layer was diluted with an equal volume of phosphate buffered saline (PBS) at a ratio of 1:1 and then slowly overlaid over the Ficoll and centrifuged for 20 minutes at 2300 rpm (1065*g*, Eppendorf AG, Germany). The white blood cell layer was removed and washed with RPMI 1640 (Invitrogen, USA) for three times and the supernatant was clear after washes. The cell pellet was re-suspended with RPMI medium containing 5% autologous plasma and 1% penicillin/streptomycin and plated onto a tissue culture Petri dish. The dish was incubated at 37°C in a humidified atmosphere with 5% CO_2 _for one hour to allow monocytes to adhere. The unattached cells were washed away by warm RPMI medium and the adherent monocytes were incubated at 37°C overnight. Subsequently, cold RPMI medium with 5 mM ethylenediaminetetraacetic acid (EDTA) was used to wash and detach the monocytes, which were finally seeded onto the tissue culture plates in RPMI medium supplemented with 5% autologous plasma and 1% penicillin/streptomycin. After 14 days of culturing, with replenishment of medium every three to four days, the monocytes derived macrophages were ready for treatment.

### Total RNA extraction and reverse transcription

Total RNA was extracted with TRIzol Reagent (Invitrogen, USA). SuperScript II transcriptase (Invitrogen, USA) was used to synthesize cDNA from the total RNA according to the manufacturer's instructions. The cDNA samples were kept at -20°C until use.

### Quantitative real-time PCR

The expression levels of IL-6, IL-8, IL-10 and TNF-α mRNA were determined with quantitative real-time PCR (q-PCR) on an ABI 7500 system using TaqMan (Applied Biosystems, USA). The relative quantification method was used to calculate the mRNA expression levels [45]. Briefly, we applied a 20 μL reaction system containing the sample cDNA, TaqMan 2× Master Mix, TaqMan probes for target genes (gene-specific Assays-on-Demand reagent kits, Applied Biosystems, USA) and TaqMan probe for 18 s rRNA as the internal control. Each sample was run in duplicates. The threshold cycle number (Ct) of the target gene was normalized to the Ct of 18 S rRNA for each sample (-ΔCt). The -ΔCt value of the mock-treated sample was subtracted from the -ΔCT value of other samples, yielding -ΔΔCt. And the value of 2^-ΔΔCt ^refers to the fold change of target gene's mRNA expression level compared to that of the corresponding mock-treated sample.

### Enzyme-linked immunosorbent assay (ELISA)

To study the RPR's differential effects on cytokines inducible by BCG, we applied ELISA to measure the amounts of IL-6, IL-8, IL-10 and TNF-α in the cell culture supernatants. Briefly, macrophages (PBMac) derived from human primary monocytes were seeded onto a 24-well plate at a concentration of 10^6^/mL. After pretreatment of PBMac with the RPR extract or its designated partially purified fractions for 16 hours, BCG (MOI = 1) was added to the cell cultures. After 24 hours of incubation, the supernatants were collected and the concentrations of cytokines were measured with ELISA according to the manufacturer's instructions (R&D Systems, USA).

### Western blot analysis

Cytoplasmic proteins and nuclear proteins were extracted separately with buffer A and buffer C as previously described [46] with modifications. Cells were washed twice with cold PBS and lysed on ice with buffer A (10 mM HEPES pH 7.9, 10 mM KCl, 0.1 mM EDTA, 0.1 mM EGTA, 1 mM DTT, 0.5 mM PMSF, 2 μg/mL aprotinin, 1 mM sodium orthovanadate, 2 μg/mL pepstatin, 2 μg/mL leupeptin and 50 mM sodium fluoride) for 15 minutes. Then 10% NP-40 was added to the cells at a final concentration of 0.625%, followed by several seconds of vortexing. The cells were then removed with a clean scraper. The lysate was then centrifuged at 15000*g *for 30 seconds. The supernatant containing cytoplasmic proteins was stored at -70°C until use. The pellet was washed gently with buffer A and centrifuged again (15000*g*, Eppendorf AG, Germany) to obtain a pellet by discarding the supernatant. The nuclear proteins were extracted by re-suspending the pellet in buffer C (20 mM HEPES pH 7.9, 0.4 M NaCl, 1 mM EDTA, 1 mM EGTA, 1 mM DTT, 1 mM PMSF, 1 μg/mL aprotinin, 1 mM sodium orthovanadate, 2 μg/mL pepstatin, 2 μg/mL leupeptin and 50 mM sodium fluoride). The sample was lysed on ice for 15 minutes with vortexing every five minutes. The nuclear extract was centrifuged (15000*g*, Eppendorf AG, Germany) at 4°C for five minutes and the supernatant was stored at -70°C until use.

Protein concentrations were quantified with the Bio-Rad protein assay kit (USA). Then an equal amount of proteins (30 μg of cytoplasmic or 4 μg of nuclear proteins) was separated by 10% SDS-PAGE and transferred to a nitrocellulose membrane. After three hours of incubation with 1% BSA in tris-buffered saline Tween-20 (TBST), the membrane was incubated with a specific antibody (1:1000 dilution) to the protein of interest for overnight at 4°C. Then the membrane was washed with TBST and incubated with the corresponding secondary antibody (1:4000 dilution) for one hour at room temperature. After five times of washes, the bands in the membrane were detected with a GE Healthcare Enhanced Chemiluminescence System (USA) according to the manufacturer's instructions. ImageJ (National Institutes of Health, USA) was used to quantify the levels of proteins captured in the laser densitometry results.

### MTT assay

MTT (3-(4,5-dimethylthiazol-2-yl)-2,5-diphenyltetrazolium bromide) assays were used to test the cytotoxicity of the RPR extract and its partially purified fractions on the cells. PBMac were seeded at a concentration of 10^6^/mL in a 24-well plate. After 48 hours of treatment with the RPR extract or its designated fractions, the cells were incubated with RPMI 1640 medium containing 0.5 mg/mL MTT (Invitrogen, USA) for one hour. Then the cell culture medium was removed and isopropanol (IPP) was added to the wells and incubated at room temperature for ten minutes. Absorbance values of the culture medium at 570 nm were measured with a microplate reader (Bio-Rad, USA).

### High performance liquid chromatography (HPLC) analysis of the extracts

The RPR extract and its fractions were dissolved in HPLC grade MeOH (Merck, Germany) before analysis. HPLC was performed with an Agilent 1200 liquid chromatography system (Agilent, USA) equipped with a reverse-phase HPLC column (Lichrospher 100, RP C_18 _EC 5 μm, 250 × 4.6 mm ID) described in our previous reports [47,48]. A gradient elution from 10% acetonitrile (CH_3_CN) to 90% CH_3_CN at a flow rate of 1 mL/min was used to separate the peaks which were detected at 210 nm with an Agilent 1200 series (Agilent, USA), a fast scanning photodiode array detector.

### Gas chromatography mass spectrometry

Before GC-MS analysis, the RPR extracts first underwent silylation. Briefly, 100 μL RPR-EA-S1 in CH_3_CN was mixed with 50 μL of pyridine and 50 μL of the derivatizing agent BSTFA [N, O-bis(trimethylsilyl)trifloroacetamide] in a 1 mL reaction vial (Alltech, USA). After incubation for two hours at 70°C, bis-trimethyl silyl trifluoroacetamide (BSTFA) replaced the labile hydrogen atom with a -Si(CH_3_)_3 _group on a wide range of polar compounds. Then the resulting mixture was analyzed by GC-MS (GC: 7890A, MS: 5975C, Agilent, USA) equipped with a HP-5MS column (Agilent, USA) (30 m × 250 μ m × 0.25 μ m). The sample (1 μl) was injected to the column. Helium was used as the carrier gas at a flow rate of 1 mL/min. The oven temperature was started at 70°C for one minute, and then increased to 180°C at a rate of 10°C/min; after two minutes' holding, increased to 280°C at a rate of 10°C/min and held for three minutes. The interface temperature was 250°C, ion source temperature was 230°C, and electron impact ionization (EI) was at 200 eV. Mass spectra were analyzed in the range of 50-700 atom mass units (amu) for a run time of 22 minutes. The G1701EA Chemstation (Agilent, USA) was used to perform the MS data analysis. The peaks of the eluants with more than 90% similarity to the compounds listed in the NIST GC-MS library were selected.

### Statistical analysis

All data were presented as the mean ± standard deviation (SD). Data were analyzed with the SPSS statistical package (version 11.5.0, SPSS Inc, USA). The statistical differences in the respective protein levels and mRNA levels among treatments were tested by one-way ANOVA followed by an Honestly Significant Difference (HSD) *post-hoc *Tukey test. *P *value of less than 0.05 is considered statistically significant.

## Results

### Extraction and bioassay guided fractionation of RPR

**Figure 2:**
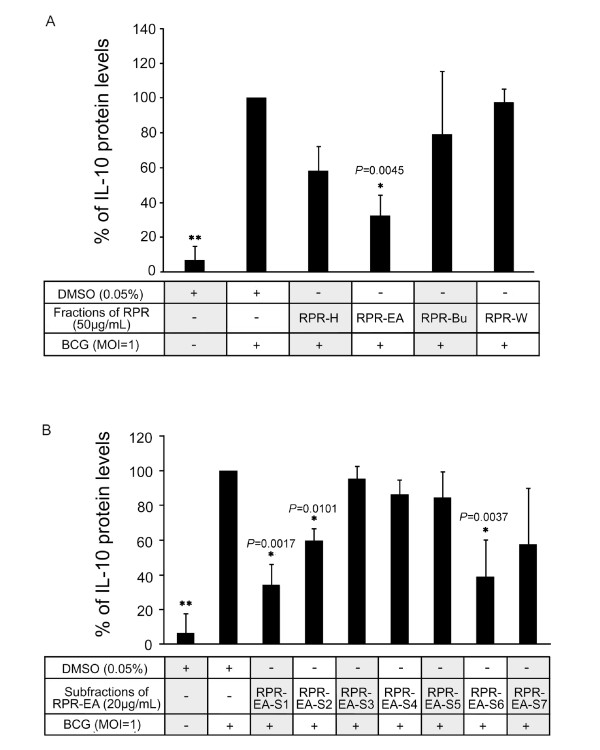
**Bioassay guided fractionation of RPR**. PBMac (5 × 10^5^) were pretreated with 0.05% DMSO or the indicated RPR fractions (A) or RPR-EA subfractions (B) overnight and then stimulated with BCG (MOI = 1). Culture supernatants were collected after 24 hours. The levels of IL-10 in the supernatants were measured by ELISA. The IL-10 concentration in the supernatant of DMSO + BCG sample was set as 100%, and the rest were compared to it to obtain a percentage value. Results are shown as mean ± SD from independent experiments on PBMac obtained from three different healthy donors. *P < 0.05, **P < 0.001 compared to the DMSO + BCG sample (one-way ANOVA, Tukey's test).

Using solvent partitioning extraction, we obtained RPR-H, RPR-EA, RPR-Bu and RPR-W. MTT assays showed that all four fractions were not toxic to PBMac at 50 μg/mL (Additional file 1). Therefore, each fraction at 50 μg/mL was tested for their inhibitory effects on BCG (MOI = 1, MOI: multiplicity of infection) induced IL-10 production in PBMac. We reported previously that 24 hours of BCG stimulation was an optimal time point for studying BCG-induced IL-10 expression [30]. The present study used the same time point. The ELISA assays found that PBMac pretreated with RPR-EA showed 68% reduction in IL-10 production induced by BCG while RPR-H, RPR-Bu and RPR-W did not show any inhibitory effect (Figure 2A). Using silica gel column chromatography, we further separated RPR-EA, the most potent fraction, into seven sub-fractions, namely RPR-EA-S1 to RPR-EA-S7 (Figure 1). Additional MTT assays demonstrated that none of them was toxic to PBMac at 50 μg/mL (Additional file 2). The ELISA results found that pretreatment of PBMac with fractions RPR-EA-S1, RPR-EA-S2 and RPR-EA-S6 (20 μg/mL) showed 66%, 40% and 62% reduction of BCG-induced IL-10 production respectively whereas the remaining fractions did not have inhibitory effects (Figure 2B). RPR-EA-S1, the most biologically active fraction, was selected for further analysis and investigation. The chemical profiles of the crude RPR extract as well as RPR-EA and RPR-EA-S1 were analyzed with reverse phase HPLC (Additional file 3).

### Differential effects of RPR extracts on BCG-induced cytokine and chemokine expression

**Figure 3:**
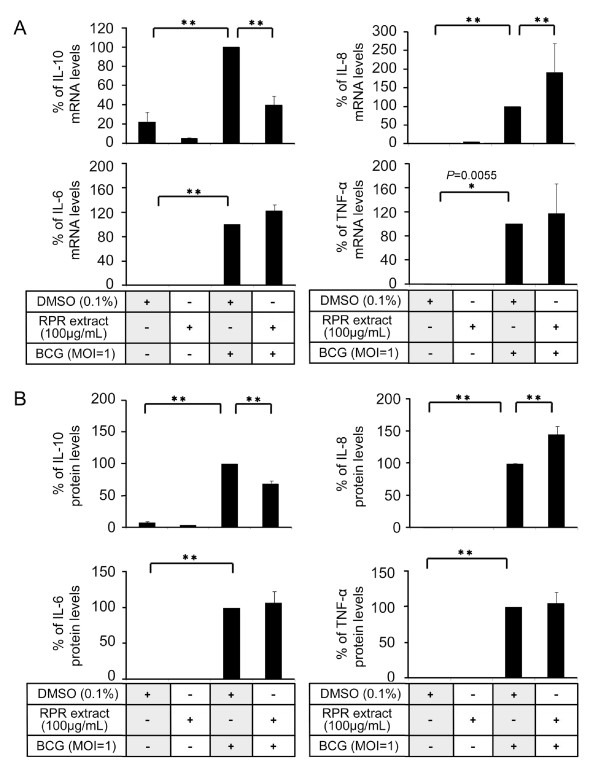
**Effects of RPR crude extract on the expression levels of cytokines in BCG-stimulated PBMac**. PBMac (5 × 10^5^) were pretreated with 0.1% DMSO or 100 μg/mL RPR extract overnight and then stimulated with BCG (MOI = 1). (A) Total mRNA was extracted after 3 hours for cDNA synthesis. The levels of mRNA were determined by q-PCR. The mRNA level of the DMSO + BCG sample was set as 100%, and the rest were compared to the DMSO + BCG sample to obtain a percentage value. (B) Culture supernatants were collected after 24 hours. The levels of IL-6, IL-8, IL-10 and TNF-α in the supernatants were measured by ELISA. The individual cytokine level in the supernatant of the DMSO + BCG sample was set as 100%, and the rest were compared to it to obtain a percentage value. Results are shown as mean ± SD from independent experiments on PBMac obtained from three (A) or six (B) different healthy donors. *P < 0.05, **P < 0.001 compared to the DMSO + BCG sample (one-way ANOVA, Tukey's test).

Since cytokines and chemokines secreted by macrophages play a critical role in the host immune responses against mycobacteria, we examined the RPR's regulatory effects on BCG-induced IL-6, IL-8, IL-10 and TNF-α expression in PBMac. Pretreatment of the immune cells with RPR extract had no effect on BCG-induced IL-6 or TNF-α expression; however, it significantly reduced the production of IL-10 and increased that of IL-8 at both mRNA and protein levels (Figure 3). The differential effects of RPR on BCG-induced cytokine and chemokine expression were further confirmed with three batches of RPR extracts (Additional file 4).

### Differential effects of RPR-EA-S1 on BCG-induced cytokine and chemokine expression

**Figure 4:**
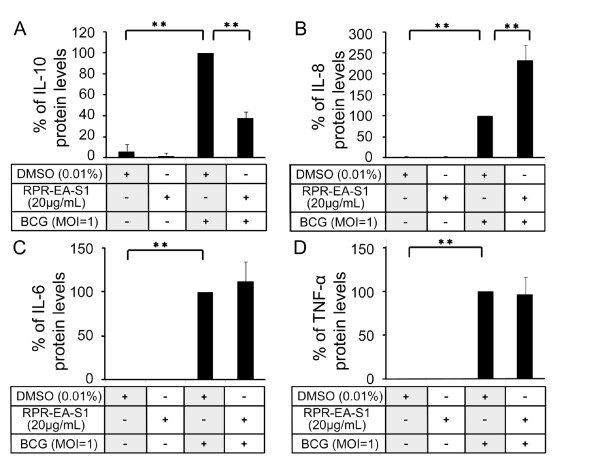
**Effects of RPR-EA-S1 on the production of different cytokines or chemokines in BCG-stimulated PBMac**. PBMac (5 × 10^5^) were pretreated with 0.01% DMSO or 20 μg/mL of RPR-EA-S1 overnight and then stimulated with BCG (MOI = 1). Supernatants were collected after 24 hours. The levels of IL-6, IL-8, IL-10 and TNF-α in the supernatants were measured by ELISA. The individual cytokine level in the supernatant of DMSO + BCG sample was set as 100%, and the rest were compared to it to obtain a percentage value. Results are shown as mean ± SD from independent experiments on PBMac obtained from four different healthy donors. **P < 0.001 compared to the DMSO + BCG sample (one-way ANOVA, Tukey's test).

To investigate whether the partially purified fraction RPR-EA-S1 contained the immuno-regulatory activity derived from the crude RPR extract, we performed ELISA on IL-6, IL-8, IL-10 and TNF-α. RPR-EA-S1's regulatory patterns on the four cytokines or chemokines were similar to those of RPR crude extracts (Figure 3 and 4). RPR-EA-S1 inhibited IL-10 and enhanced IL-8 expression, while had no effect on IL-6 and TNF-α expression in the BCG-induced PBMac. When compared to the crude RPR extract (Figure 3B), RPR-EA-S1 was more effective in terms of the levels of IL-10 inhibition and IL-8 induction in BCG-stimulated PBMac (Figure 4).

**Figure 5:**
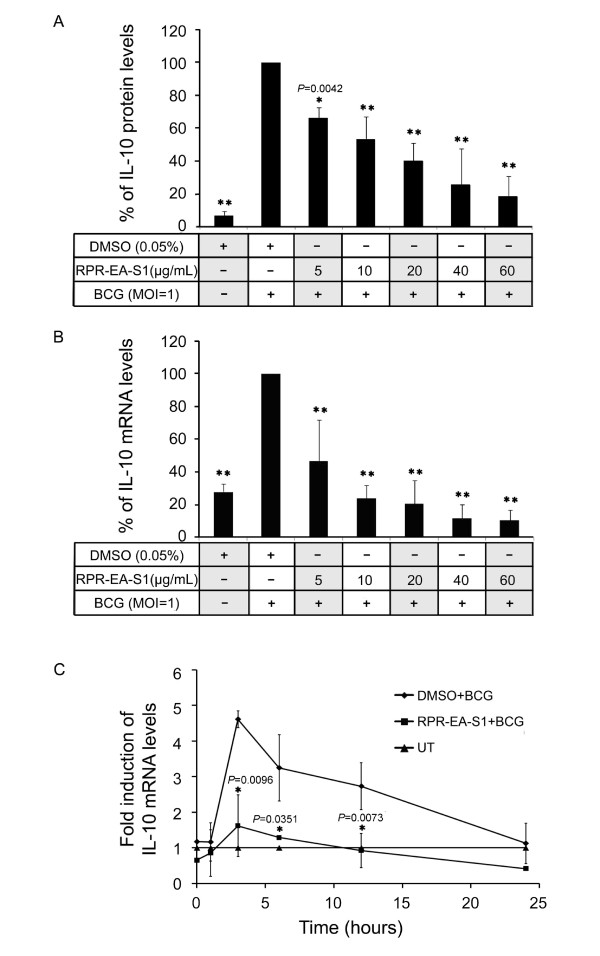
**Dose-dependent and time course effects of RPR-EA-S1 on the production of IL-10 in BCG-stimulated PBMac**. PBMac (5 × 10^5^) were pretreated with 0.05% DMSO or different dose of RPR-EA-S1 overnight and then stimulated with BCG (MOI = 1). (A) Supernatants were collected after 24 hours. The levels of IL-10 in the supernatants were measured by ELISA. (B) Total mRNA was extracted after 3 hours for cDNA synthesis. The levels of mRNA were determined by q-PCR. The mRNA or protein expression levels of DMSO + BCG sample were set as 100%, and the rest were compared to DMSO + BCG to obtain a percentage value. (C) PBMac (5 × 10^5^) were pretreated with 0.01% DMSO or 20 μg/mL of RPR-EA-S1 overnight and then stimulated with BCG (MOI = 1) for 0, 1, 3, 6, 12 and 24 hours. Total mRNA was used to synthesize cDNA. The levels of IL-10 mRNA were determined by q-PCR. Results are shown as mean ± SD from independent experiments on PBMac obtained from three different healthy donors. *P < 0.05, **P < 0.0001 compared to the DMSO + BCG sample (one-way ANOVA, Tukey's test for Figures 5A and B, t-test for Figure 5C).

Moreover, the inhibitory effect of RPR-EA-S1 on BCG-induced IL-10 production was in a dose-dependent manner between 5 μg/mL and 50 μg/mL both at the protein and mRNA levels (Figure 5A and 5B). The regulatory effects were significant even at a low concentration of 5 μg/mL.

The IL-10 mRNA expression levels were studied over a time course of 24 hours. Figure 5C shows that the BCG-induced IL-10 expression reached the peak level at three hours before declining to the basal level at 24 hours; however, pretreatment of the cells with RPR-EA-S1 inhibited BCG-induced IL-10 mRNA expression throughout the time course.

RPR-EA-S1, a fraction of RPR, demonstrated differential effects on cytokine and chemokine expression in BCG-stimulated PBMac, enhanced BCG-induced IL-8 production and inhibited IL-10 production in a dose and time-dependent manner.

### Mechanisms underlying RPR-EA-S1 inhibition of IL-10 expression in BCG-stimulated blood macrophages

**Figure 6:**
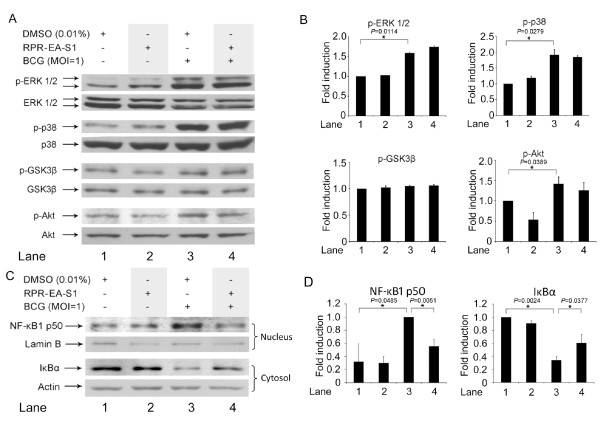
**Effects of RPR-EA-S1 on signaling kinases and NF-κB expression in BCG-stimulated PBMac**. PBMac (2 × 10^6^) were pretreated with 0.01% DMSO or 20 μg/mL of RPR-EA-S1 overnight and then stimulated with BCG (MOI = 1) for 30 minutes. Cytoplasmic proteins and nuclear proteins were extracted separately. (A) and (C): The levels of phospho-MAPK, phospho-GSK and phospho-Akt, as well as NF-κB1 p50 and IκBα were analyzed with Western blot. The figures showed one set of representative results from independent experiments on PBMac obtained from three different healthy donors. (B) and (D): Quantification of the Western blot was measured by laser densitometry. Intensities of phospho-MAPK, phospho-GSK, phospho-Akt, NF-κB1 p50 and IκBα were normalized to the corresponding MAPK, GSK, Akt, Lamin B and Actin, respectively. Results are shown as mean ± SD from independent experiments on PBMac obtained from three different healthy donors. *P < 0.05 compared to the DMSO + BCG sample (one-way ANOVA, Tukey's test).

After confirming the inhibitory effect of RPR-EA-S1 on BCG-induced IL-10 expression, we investigated the mechanisms underlying the decrease of IL-10 expression. Mitogen-activated protein kinases (MAPK) are involved in IL-10 regulation [3,25]. BCG increases the phosphorylation of ERK1/2 and p38 [3]. This study showed that pretreatment with RPR-EA-S1 did not affect the phosphorylation levels of ERK1/2 and p38 induced by BCG stimulation (Figure 6A and 6B). Since Akt/GSK3β is a key pathway involved in BCG-induced IL-10 expression [30], we examined the effect of RPR-EA-S1 on this pathway. The study found that the phosphorylation of Akt or GSK3β, inducible by BCG, was not affected by pretreatment of the cells with RPR-EA-S1 (Figure 6A and 6B). Neither MAPK kinases nor the Akt/GSK3β pathway was involved in the inhibitory effects of RPR on BCG-induced IL-10 production.

Downstream of these kinases, specific transcriptional factors may also affect IL-10 expression. Our previous study demonstrated a correlation between the translocation of NF-κB1 p50 into the nucleus and the induction of IL-10 by BCG [30]. In the present study, Western blotting was performed to examine the translocation of NF-κB1 p50 to the nucleus. The results confirmed the induced translocation of NF-κB1 p50 by BCG while RPR-EA-S1 pretreatment of the cells reduced such effects (Figure 6C and 6D). Similarly, the IκBα was degraded after BCG stimulation while pretreatment of the cells with RPR-EA-S1 significantly inhibited this degradation (Figure 6C and 6D).

**Figure 7:**
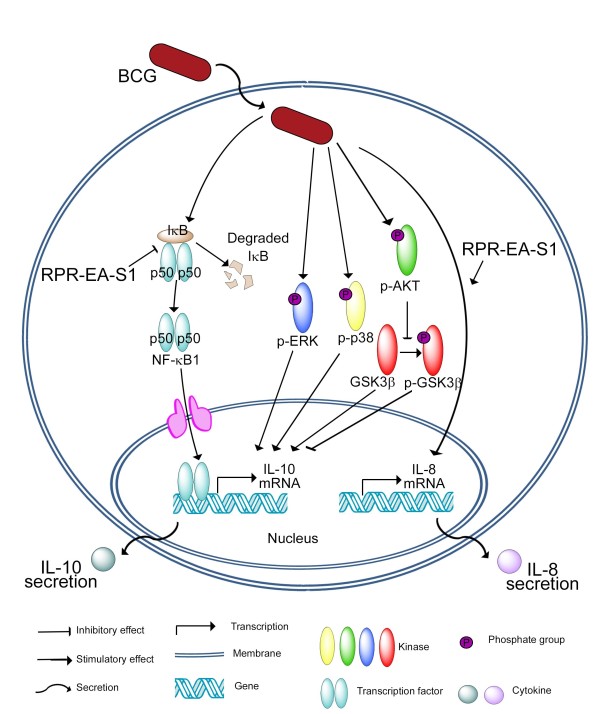
**Mechanisms underlying the immunomodulation effects of RPR-EA-S1**. In BCG-stimulated macrophages, RPR-EA-S1 can specifically inhibit IL-10 production and yet enhance IL-8 expression. RPR-EA-S1 did not affect the phosphorylation of cellular protein kinases including ERK, p38, Akt and GSK3β. Its inhibitory effects in BCG-activated IL-10 expression may be, at least in part, through the suppression of the degradation of IκBα in the cytoplasm and thus inhibited the translocation of transcription factor NF-κB1 p50 to the nucleus.

Suppression of BCG-induced IL-10 by RPR-EA-S1 were achieved through suppression of IκBα degradation and inhibition of NF-κB1 p50 translocation to the nucleus (Figure 7).

### GC-MS analysis of the chemical constituents of RPR-EA-S1

To further investigate the chemical components of RPR-EA-S1, we performed GC-MS analysis (Additional files 5 and 6). The mass spectra generated from the extracts were compared to the compounds in NIST GC-MS library. Two major groups of compounds were found in RPR-EA-S1, namely phenols (including hydroquinone, 4-hydroxybenzoic acid and ferulic acid) and fatty acids (including unsaturated and saturated fatty acids).

## Discussion

Recent resurgence of mycobacterial infection is in part due to the spread of AIDS in developing countries. Combined administration of specific anti-mycobacterial drugs is the current TB treatment recommended by the World Health Organization [49]. With increases in multidrug-resistant-TB (MDR-TB) and extensively drug-resistant (XDR)-TB strains, the need to develop new anti-Mtb drugs is urgent and immuno-modulatory agents have been considered to enhance TB treatment [50].

We demonstrated that the crude RPR extract and its fraction RPR-EA-S1 augmented BCG-induced IL-8 expression (Figure 3 and 4). Decreased IL-8 secretion was observed in HIV-infected patients with miliary TB [20]. *In vivo *studies showed that anti-IL-8 antibody inhibited the formation of granuloma in rabbits [51], suggesting a significant role for IL-8 in controlling Mtb infection by the host immunity. Recent studies showed that IL-8 attracted neutrophils and T lymphocytes to the infection sites for the formation of granuloma and augmentation of cell mediated immunity [52]. IL-8 also activated neutrophils to exhibit bactericidal responses [53]. Therefore, the enhanced IL-8 induction by RPR may be beneficial to the initiation of host immune responses against Mtb and the subsequent formation of the granuloma in human [51].

We also showed that crude RPR extract and its fraction RPR-EA-S1 can inhibit BCG-induced IL-10 expression (Figure 3, 4, 5). IL-10 is a well known anti-inflammatory cytokine produced by macrophages and T cells during Mtb infection [3]. The relationship between IL-10 production and TB progression was well observed in both animal and human studies [32,33,36]. Recent studies revealed that Mtb utilized IL-10 to evade the host immunity. Firstly, induction of IL-10 inhibited the production of bactericidal molecules including NO and RNIs by macrophages [54]. Secondly, IL-10 suppressed pro-inflammatory cytokines such as IL-12, TNF-α and IFN-γ, resulting in reduced Th1 cell immunity, CD4+ and CD8+ lytic activity, as well as delayed macrophage activation [31]. Thirdly, IL-10 suppressed antigen presentation by down-regulating MHC and other co-stimulatory molecules on the cell surface of monocytes/macrophages [31]. We recently showed that this effect was due to IL-10 activation of STAT3 to suppress cathepsin S expression [29]. Fourthly, IL-10 inhibited TNF-α activity by down-regulating TNF-α expression and inducing soluble TNF receptor 2, leading to reduced apoptosis and increased pathogen survival [55]. Fifthly, macrophage-derived IL-10 triggered alternative macrophage activation in Mtb infection, in which arginase-1 gene expression was strikingly enhanced. In turn, arginase-1 negatively regulated microbicidal mechanisms in macrophages and promotes Mtb recrudescence [56]. The inhibitory effects of RPR on BCG-induced IL-10 production may have a beneficial effect on the host for the reversal of the immunosuppressive effects of IL-10 thereby speeding up the killing of Mtb.

Multiple pathways are involved in the regulation of BCG-induced IL-10 expression, including ERK1/2, p38 and PI3K/GSK3 [3,30]. In the present study, RPR showed no effect on either of these pathways (Figure 6A and 6B); however, RPR inhibited NF-κB1 p50 translocation to the nucleus (Figure 6C and 6D). NF-κB plays a role in promoting the production of cytokines [57]. NF-κB is a family of transcriptional factors, including RelA (p65), NF-κB1 (p50 and p105), NF-κB2 (p52 and p100), c-Rel and RelB, bound with an inhibitory protein named IκB in the cytoplasm. After stimulation, IκB undergoes phosphorylation and degradation, resulting in the release of NF-κB for translocation to the nucleus, leading to transcription of their regulated target genes [58]. Among members of the NF-κB family, NF-κB1 p50/p50 homodimer was involved in IL-10 transcription [59]. In murine macrophages, NF-κB1 p50/p50 homodimer activated transcription of IL-10 together with the CREB protein [59]. Macrophages from NF-κB1 p50 knock-out mice expressed less IL-10 compared to normal mice upon lipopolysaccharide challenge [59]. The present study shows that RPR-EA-S1 significantly reduced the degradation of IκBα in the cytoplasm and inhibited the translocation of NF-κB1 p50 to the nucleus in BCG-induced human blood macrophages (Figure 6). Therefore, the inhibition of IL-10 by RPR-EA-S1 may be mediated by the effects of its constituents on NF-κB1 translocation (Figure 7).

While NF-κB pathway also regulates IL-6, IL-8 and TNF-α, previous studies indicated that the transcriptions of IL-6, IL-8 and TNF-α were not mainly regulated by NF-κB1 p50 homodimer [59-61]. Instead, silencing of NF-κB p65 rather than p50 gene was effective in down-regulating TNF-α induced IL-6 and IL-8 mRNA synthesis [61]. In luciferase activity assays of the TNF-α promoter, p50 plays little role in driving the transcription, while c-Rel and p65 plays dominant roles in inducing the transcription [60]. Therefore, the inhibition of NF-κB1 p50 translocation to the nucleus cannot affect BCG-induced IL-6, IL-8 and TNF-α expression. Moreover, the transcriptional regulation of cytokines involved multiple transcriptional factors and was inducer-dependent [62-64]. For example, respiratory syncytial virus (RSV) may increase IL-8 production in the airway epithelium partly via the activation of transcription factors including NF-κB and NF-IL-6 [65] whereas AP-1 and NF-κB are essential transcription factors for IL-1β-induced IL-8 gene expression [66]. In BCG stimulated macrophages, it is possible that in addition to NF-κB, multiple transcriptional factors may contribute to the expression of IL-6, IL-8 and TNF-α. The inhibition of NF-κB pathway may be compensated by other transcriptional factors. This may also explain why the BCG-induced IL-6, IL-8 and TNF-α expression were not inhibited by RPR-EA-S1.

The major groups of compounds in the RPR-EA-S1 fraction were phenols and fatty acids. Both phenols and fatty acids are biologically active compounds commonly present in the genus of *Paeonia *[67-69]. For example, ferulic acid, one of the phenols isolated from *Paeonia*, scavenges free reactive radicals as determined by DPPH (1, 1-Diphenyl-2-picrylhydrazyl) assay and inhibits lipid peroxidation and protects DNA from oxidative damage [69]. Phenylacetic acid, plant hormone that had been used to treat type II hyperammonemia [70], was also detected in RPR-EA-S1. Our future studies will isolate the compounds responsible for the immuno-regulatory effects of RPR-EA-S1.

## Conclusion

RPR crude extracts and its fraction RPR-EA-S1 specifically inhibited anti-inflammatory cytokine IL-10 and enhanced pro-inflammatory chemokine IL-8 expression in BCG-activated PBMac. The inhibitory effects of RPR-EA-S1 on IL-10 expression in BCG-activated PBMac may be due to the reduced nuclear translocation of NF-κB1 p50.

## Abbreviations

amu: atom mass units; BCG: Bacillus Calmette-Guerin; BSTFA: bis-trimethyl silyl trifluoroacetamide; CH_3_CN: acetonitrile; Ct: threshold cycle number; DCs: dendritic cells; EDTA: ethylenediaminetetraacetic acid; EI: electron impact ionization; ELISA: enzyme-linked immunosorbent assay; EtOAc: ethyl acetate; EtOH: ethanol; GC-MS: gas chromatography mass spectrometry; GSK3β: glycogen synthase kinase-3β; HIV: human immunodeficiency virus; HO-1: heme oxygenase-1; HPLC: high performance liquid chromatography; HSD: Honestly Significant Difference; IFN-γ: interferon-γ; IL-6: interleukin-6; IL-8: interleukin-8; IL-10: interleukin-10; iNOS: inducible nitric oxide synthases; IPP: isopropanol; MAPK: mitogen-activated protein kinases; MDR-TB: multidrug-resistant TB; MeOH: methanol; MOI: multiplicity of infection; Mtb: Mycobacterium tuberculosis; MTT: 3-(4,5-dimethylthiazol-2-yl)-2,5-diphenyltetrazolium bromide; *n*-BuOH: *n*-butanol; *n*-C_6_H_14_: hexane; NF-κB1 p50: nuclear factor-κB1 p50; NO: nitric oxide; PBMac: peripheral blood monocytes-derived macrophages; PBS: phosphate buffered saline; q-PCR: quantitative real-time PCR; RNIs: reactive nitrogen intermediates; ROIs: reactive oxygen intermediates; RPR: Radix Paeoniae Rubra; RSV: respiratory syncytial virus; SD: standard deviation; TB: tuberculosis; TBST: tris-buffered saline; Tween-20; TNF-α: tumor necrosis factor alpha; XDR-TB: extensively drug-resistant TB

## Competing interests

The authors declare that they have no competing interests.

## Authors' contributions

LW designed and performed the experiments, analyzed the data and wrote the manuscript. CLY, JCL and TCO helped design the study, interpreted the data and revised the manuscript. GC and JZ performed the initial extraction and partial purification of the herb. ASL conceived the idea of this study, designed the experiments, supervised the team and revised the manuscript. All authors read and approved the final version of the manuscript.

## Supplementary Material

Additional file 1 **Effects of different RPR fractions on the cell viability of human PBMac**. PBMac (5 × 10^5^) were treated with different doses of RPR fractions for 48 hours. The cell viability was tested by MTT assay. Results are shown as mean ± SD from independent experiments on PBMac obtained from three different healthy donors. * *P *< 0.05 compared to the DMSO treated sample (one-way ANOVA, Tukey's test). Click here for file

Additional file 2 **Effects of different RPR-EA subfractions on the cell viability of human PBMac**. PBMac (5 × 10^5^) were treated with different doses of extracts for 48 hours. The cell viability was tested by MTT assay. Results are shown as mean ± SD from independent experiments on PBMac obtained from three different healthy donors. Click here for file

Additional file 3 **HPLC chromatogram of RPR, RPR-EA and RPR-EA-S1**. The HPLC was performed by using a reverse-phase HPLC column (Lichrospher 100 RP C18 EC 5 μm, 250 × 4.6 mm ID) and the detection wavelength (WL) was set at 210 nm. The flow rate was 1 mL/min. The solvents used in gradient elution were (A) water and (B) acetonitrile (CH_3_CN). The program was set as follows.
Time (Minutes)   % Solvent (A)   % Solvent (B)
0   90   10
10   60   40   Gradient
15   10   90   Gradient
17   10   90   Isocratic
20   90   10   Gradient
25   90   10   Isocratic Click here for file

Additional file 4 **Effects of three different batches of RPR extracts on cytokine expression in BCG-stimulated PBMac**. PBMac (5 × 10^5^) were pretreated with 0.1% DMSO or 100 μg/mL of different batches of RPR extracts (R1, R2 and R3) overnight and then stimulated with BCG (MOI = 1). Supernatants were collected after 24 hours. The levels of IL-6, IL-8, IL-10 and TNF-α in the supernatants were measured by ELISA. The individual cytokine level in the supernatant of the DMSO + BCG sample was set as 100%, and the rest were compared to it to obtain a percentage value. Results are shown as mean ± SD from independent experiments on PBMac obtained from three different healthy donors. *p < 0.05, ** *p *< 0.001 compared to the DMSO + BCG sample (one-way ANOVA, Tukey's test). Click here for file

Additional file 5 **total ion chromatogram of RPR-EA-S1**. After reaction with derivatizing agent BSTFA [N, O-bis (trimethylsilyl) trifloroacetamide], RPR-EA-S1 was analyzed by GC-MS equipped with a HP-5MS column (30 m × 250 mm × 0.25 mm). The spectra of the peaks were compared to the spectra listed in the NIST GC-MS library. Only peaks with >90% similarity to the compounds in database were listed in Additional file 6. Click here for file

Additional file 6 **Compounds detected in RPR-EA-S1 using GC-MS**. Click here for file
