# Supplementary figures and images for: Differential effects of Radix Paeoniae Rubra (Chishao) on cytokine and chemokine expression inducible by mycobacteria
Source: Chin Med. 2011 Mar 30;6:14. doi: 10.1186/1749-8546-6-14 (PMC3076300; doi:10.1186/1749-8546-6-14)

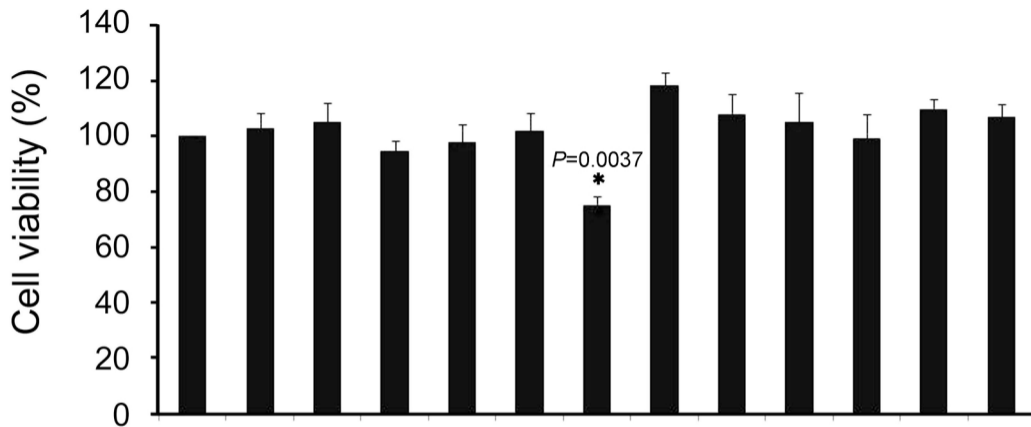

|                  |   |       |    |     |        |    |     |        |    |     |       |    |     |
|------------------|---|-------|----|-----|--------|----|-----|--------|----|-----|-------|----|-----|
| DMSO (0.1%)      | + | -     | -  | -   | -      | -  | -   | -      | -  | -   | -     | -  | -   |
| Dose (µg/mL)     | - | 20    | 50 | 100 | 20     | 50 | 100 | 20     | 50 | 100 | 20    | 50 | 100 |
| Fractions of RPR | - | RPR-H |    |     | RPR-EA |    |     | RPR-Bu |    |     | RPR-W |    |     |

Supplement: Additional file 1 — Effects of different RPR fractions on the cell viability of human PBMac. PBMac (5 × 10^5^) were treated with different doses of RPR fractions for 48 hours. The cell viability was tested by MTT assay. Results are shown as mean ± SD from independent experiments on PBMac obtained from three different healthy donors. * P < 0.05 compared to the DMSO treated sample (one-way ANOVA, Tukey's test). [file 1749-8546-6-14-S1.PDF]

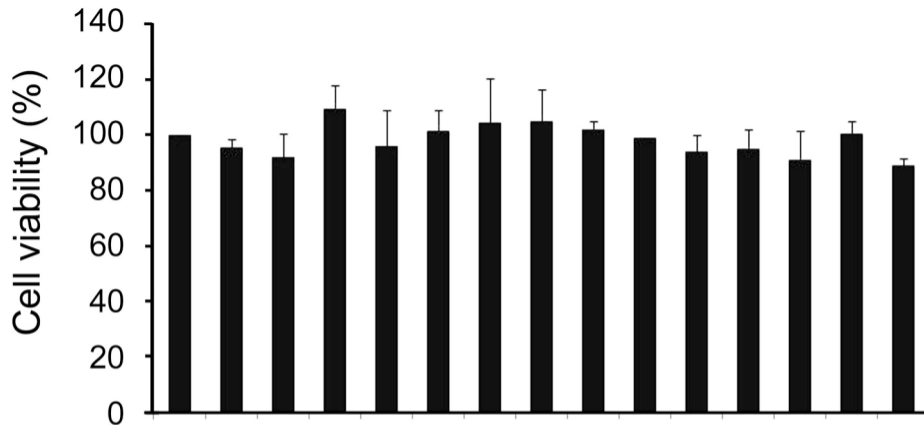

|                        |   |           |    |           |    |           |    |           |    |           |    |           |    |           |    |
|------------------------|---|-----------|----|-----------|----|-----------|----|-----------|----|-----------|----|-----------|----|-----------|----|
| DMSO (0.1%)            | + | -         | -  | -         | -  | -         | -  | -         | -  | -         | -  | -         | -  | -         | -  |
| Dose (µg/mL)           | - | 20        | 50 | 20        | 50 | 20        | 50 | 20        | 50 | 20        | 50 | 20        | 50 | 20        | 50 |
| Subfractions of RPR-EA | - | RPR-EA-S1 |    | RPR-EA-S2 |    | RPR-EA-S3 |    | RPR-EA-S4 |    | RPR-EA-S5 |    | RPR-EA-S6 |    | RPR-EA-S7 |    |

Supplement: Additional file 2 — Effects of different RPR-EA subfractions on the cell viability of human PBMac. PBMac (5 × 10^5^) were treated with different doses of extracts for 48 hours. The cell viability was tested by MTT assay. Results are shown as mean ± SD from independent experiments on PBMac obtained from three different healthy donors. [file 1749-8546-6-14-S2.PDF]

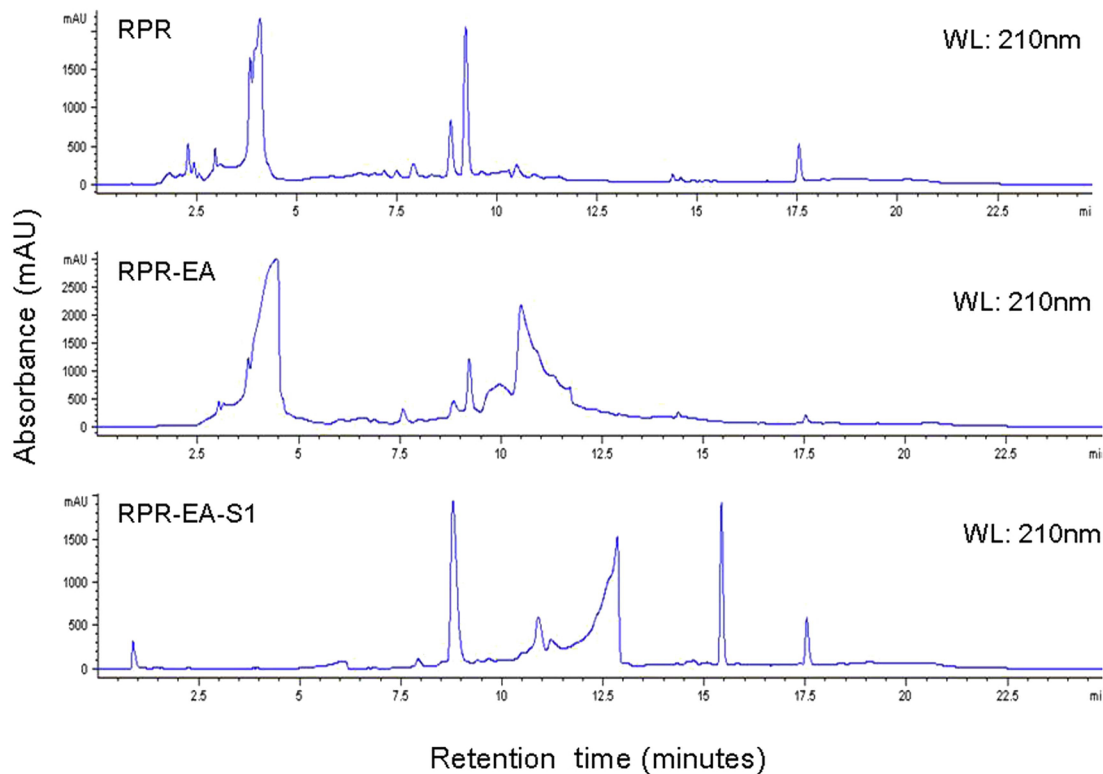

Supplement: Additional file 3 — HPLC chromatogram of RPR, RPR-EA and RPR-EA-S1. The HPLC was performed by using a reverse-phase HPLC column (Lichrospher 100 RP C18 EC 5 μm, 250 × 4.6 mm ID) and the detection wavelength (WL) was set at 210 nm. The flow rate was 1 mL/min. The solvents used in gradient elution were (A) water and (B) acetonitrile (CH3CN). The program was set as follows. Time (Minutes) % Solvent (A) % Solvent (B) 0 90 10 10 60 40 Gradient 15 10 90 Gradient 17 10 90 Isocratic 20 90 10 Gradient 25 90 10 Isocratic [file 1749-8546-6-14-S3.PDF]

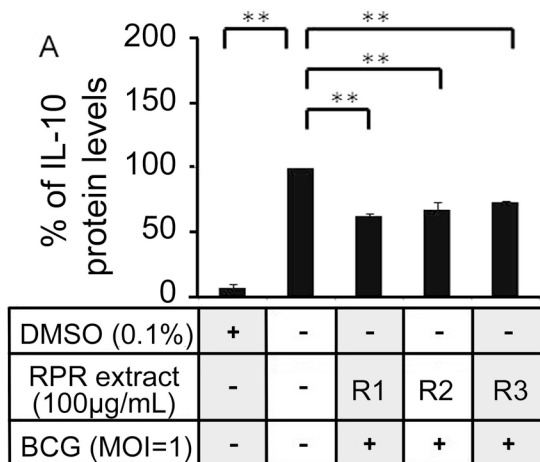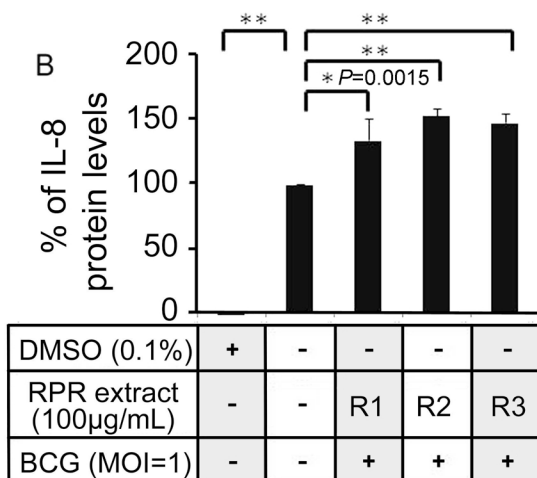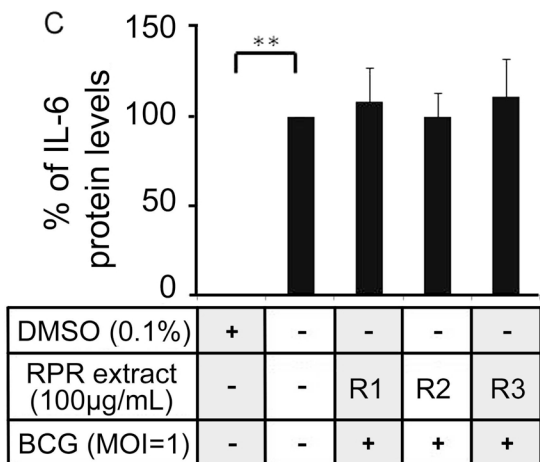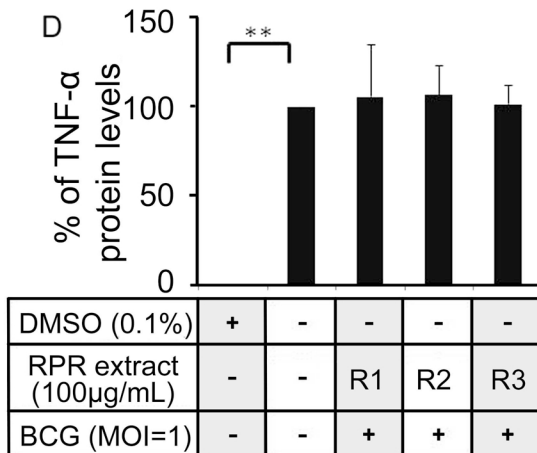

Supplement: Additional file 4 — Effects of three different batches of RPR extracts on cytokine expression in BCG-stimulated PBMac. PBMac (5 × 10^5^) were pretreated with 0.1% DMSO or 100 μg/mL of different batches of RPR extracts (R1, R2 and R3) overnight and then stimulated with BCG (MOI = 1). Supernatants were collected after 24 hours. The levels of IL-6, IL-8, IL-10 and TNF-α in the supernatants were measured by ELISA. The individual cytokine level in the supernatant of the DMSO + BCG sample was set as 100%, and the rest were compared to it to obtain a percentage value. Results are shown as mean ± SD from independent experiments on PBMac obtained from three different healthy donors. *p < 0.05, ** p < 0.001 compared to the DMSO + BCG sample (one-way ANOVA, Tukey's test). [file 1749-8546-6-14-S4.PDF]

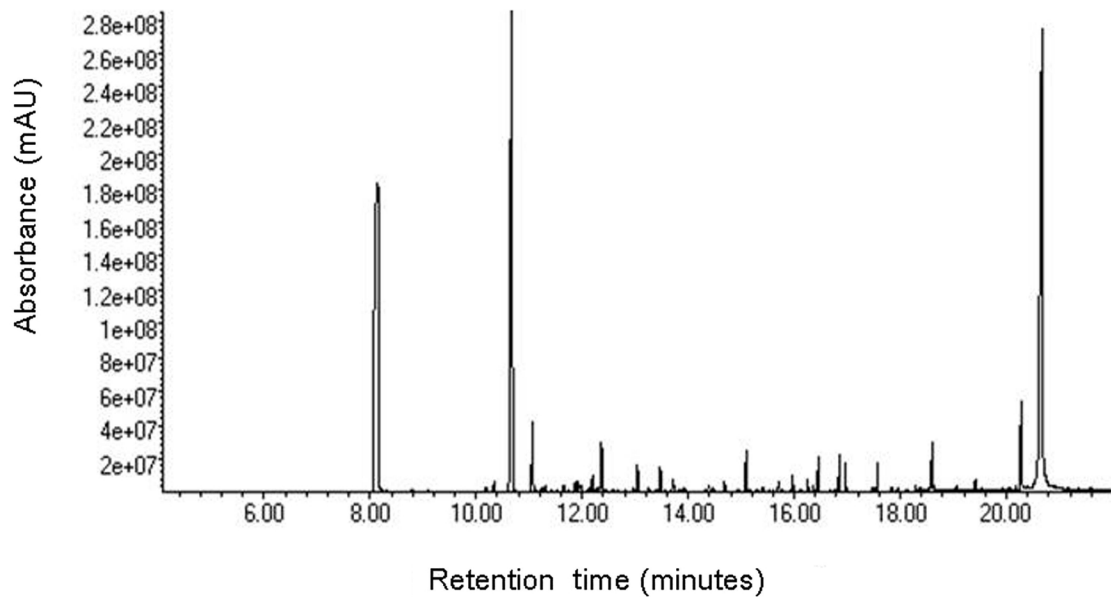

Supplement: Additional file 5 — total ion chromatogram of RPR-EA-S1. After reaction with derivatizing agent BSTFA [N, O-bis (trimethylsilyl) trifloroacetamide], RPR-EA-S1 was analyzed by GC-MS equipped with a HP-5MS column (30 m × 250 mm × 0.25 mm). The spectra of the peaks were compared to the spectra listed in the NIST GC-MS library. Only peaks with >90% similarity to the compounds in database were listed in Additional file 6. [file 1749-8546-6-14-S5.PDF]
