# Supplementary material for: Differential effects of Radix Paeoniae Rubra (Chishao) on cytokine and chemokine expression inducible by mycobacteria
Source: Chin Med. 2011 Mar 30;6:14. doi: 10.1186/1749-8546-6-14 (PMC3076300; doi:10.1186/1749-8546-6-14)
Supplement: Additional file 6 — Compounds detected in RPR-EA-S1 using GC-MS. [file 1749-8546-6-14-S6.PDF]

Compounds	Retention time	% similarity	Name	Structure
1	10.184	96	hydroquinone	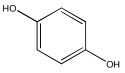
2	11.875	96	4-Hydroxy-3-methoxy-benzaldehyde	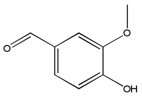
3	12.301	97	4-(2-hydroxyethyl)phenol	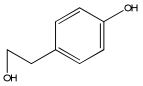
4	12.586	94	4-(1-hydroxyvinyl)phenol	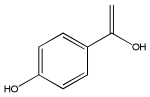
5	13.042	94	4-hydroxybenzoic acid	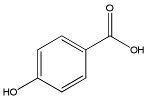
6	13.042	90	3-hydroxybenzoic acid	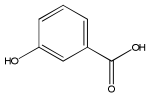
7	15.097	96	4-hydroxy-3-methoxybenzoic acid	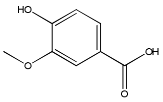
8	16.768	96	(E)-3-(4-hydroxyphenyl)acrylic acid	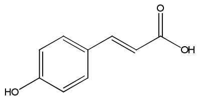
9	17.842	99	(E)-3-(3-hydroxy-4-methoxyphenyl)acrylic acid	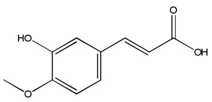
10	17.842	94	Ferulic acid	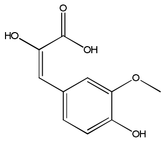
11	17.842	93	(E)-3-(4-hydroxy-3-methoxyphenyl)acrylic acid	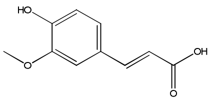
12	18.48	96	(9Z,12Z)-octadeca-9,12-dienoic acid	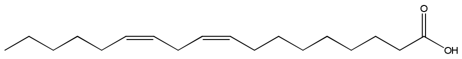
13	18.506	96	(Z)-octadec-11-enoic acid	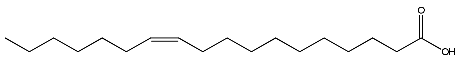

Compounds	Retention time	% similarity	Name	Structure
14	13.317	90	dodecanoic acid	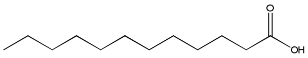
15	15.912	95	tetradecanoic acid	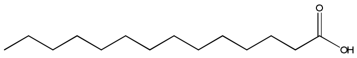
16	17.479	98	palmitic acid	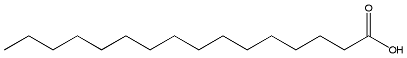
17	18.631	98	stearic acid	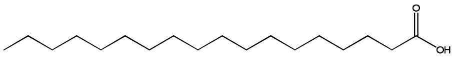
18	5.358	94	cyclohex-1-enol	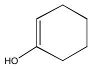
19	8.788	93	2-phenylacetic acid	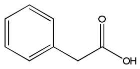
20	11.839	90	5-(4-chlorophenyl)-1H-1,2,4-triazole-3-carbaldehyde	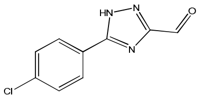
21	16.965	95	(E)-ethyl 3,7,11,15-tetramethylhexadec-2-enoate	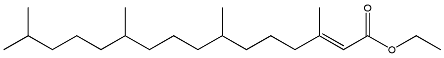
22	16.171	91	ethyl 4-acetyl-3-(3-ethoxy-3-oxopropyl)-5-methyl-1H-pyrrole-2-carboxylate	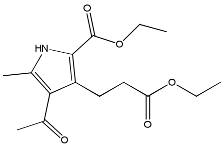
23	19.414	95	Hexanedioic acid, bis(2-ethylhexyl) ester	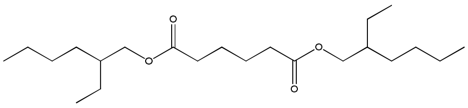
